# The effect of date palm on sexual function in infertile couples: a double-blind controlled clinical trial

**DOI:** 10.1186/s13104-022-05945-0

**Published:** 2022-02-15

**Authors:** Athar Rasekh Jahromi, Zahra Mosallanezhad, Fatemeh Saadat Hosini, Safieh Jamali, Nader Sharifi

**Affiliations:** 1 Department of Gynecology & Obstetrics, Jahrom University of Medical Sciences, Jahrom, Iran; 2 Student Research Committee, Jahrom University of Medical Sciences, Jahrom, Iran; 3 Research Center for Social Determinants of Health, Jahrom University of Medical Sciences, Main Campus, Motahari Boulevard, Jahrom, 7414846199 Iran; 4 Department of Public Health, Khomein University of Medical Sciences, Khomein, Iran

**Keywords:** Infertility, Date palm, Sexual function, Female sexual function index, Male sexual function index

## Abstract

**Objective:**

Infertility has a significant impact on the sexual function of couples. The use of herbal medicine has been highly important throughout the history of medicine. The present study was conducted to evaluate the effect of date palm on sexual function of infertile couples.

**Results:**

The present study was a double-blind, placebo-controlled clinical trial conducted on infertile women and their husbands who referred to infertility clinics in Iran in 2019. The intervention group was given a palm date capsule and the control group was given a placebo. Data were collected through female sexual function index and International Index of Erectile Function. The total score of sexual function of females in the intervention group increased significantly from 21.06 ± 2.58 to 27.31 ± 2.59 (P < 0.0001). Also, other areas of sexual function in females (arousal, orgasm, lubrication, pain during intercourse, satisfaction) in the intervention group showed a significant increase compared to females in the control group, which was statistically significant (P < 0.0001). All areas of male sexual function (erectile function, orgasmic function, sexual desire, intercourse satisfaction and overall satisfaction) significantly increased in the intervention group compared to the control group (P < 0.0001). The present study revealed that 1-month consumption of date palm has a positive impact on the sexual function of infertile couples.

*Trial registration* The trial was retrospectively registered in the Iranian registry of clinical trials at 2020-10-07 (https://www.irct.ir/trial/51339; registration number: IRCT20200925048834N1)

## Introduction

Infertility refers to inability of couples to be fertile after 1 year without using contraceptive methods [1, 2]. Fifteen percent of women of childbearing age are infertile [3]. It is estimated that 10% of population, 13% of women, 10% of men, and 15% of couples around the world to be infertile [4, 5]. Results of a meta-analysis study in Iran revealed that the prevalence of lifetime primary infertility, current primary infertility, and current secondary infertility is 13.96%, 3.09%, and 2.18%, respectively [6]. Infertility is not only a medical problem, but also affects all personal dimensions and social life of most of infertile individuals. Infertile couples are more prone to psychological problems (anxiety, depression, and stress), which may result in marital distress, social dysfunction (stigma, social exclusion, and feelings of failure), and reduce the quality of life [7–9]. Infertility and infertility management affects different dimensions of a couple's life. Sexual dysfunctions can appear in both partners and might provoke problems in every stage of sexual response [10]. Infertility negatively affects the sexuality of infertile couples. Numerous studies show that infertile women have lower sexual function than fertile women [11–13]. Sexual satisfaction is strongly affected by the consequences of infertility such as reduced self-esteem, feelings of depression and anxiety, and failed sexual relationship [14, 15]. Use of herbal medicine has played an important role throughout the history of medicine, and human beings have always used plants to treat their diseases throughout history. Herbal medicine is used to treat infertility in traditional medicine. Date is one of the herbal medicines that have important pharmacological effects. Egyptian scientists have reported that date palm can stimulate the gonad [16]. Valid books in traditional medicine have referred to several medicinal properties of date palm, including strengthening the sexual desire of males and increasing lust in females [17]. Also, the presence of compounds such as alkaloids, saponins and flavonoids in dates increases sexual desire and sexual intercourse in males and females by affecting the central nervous system and stimulating dopamine secretion, activating the mesolimbic system and the *nucleus accumbens* [18]*.*

Since ancient times, date palm has been used in Greece, China and Egypt to treat infertility and increase sexual desire and fertility in females [19]. Rasekh indicated that Palm Pollen is effective in sperm parameters of infertile men [20]. Administering date palm to male rats and measuring the sexual parameters of rats showed an improvement in their sexual function [18]. There are few studies on the effect of date palm on male and female sexual function in human beings. Studies on animals have shown its effect on the parameters of semen analysis in male animals and increasing hormones. Moreover, there are sexual problems in infertile couples and females tend to complementary medicine alternative therapies. Hence, the purpose of the present study was to investigate the effect of date palm on sexual function of infertile couples.

## Main text

### Methods and materials

#### Study design

The present study was a double-blind, randomized, placebo-controlled trial to assess the effect of date palm on sexual function in infertile couples. This study was conducted on 128 infertile couples (64 couples in the intervention and 64 couples in the placebo groups) who referred to the infertility centers affiliated with the Jahrom University of Medical Sciences, Jahrom, Iran, from December 22, 2019, to March 15, 2020. The study was started after obtaining written consent from the patients, explaining the objectives of the project and obtaining an ethics permission from the Ethics Council of Jahrom University of Medical Sciences (under the code of ethics of IR.JUMS.RES.1398.049 and retrospectively registered it in Iranian Registry of Clinical Trials under the code of IRCT 20200925048834N1 (https://www.irct.ir/trial/51339)).

#### Participants

We recruited samples aged 15–49 years old. Inclusion criteria were informed consent, proved infertility (not being pregnant after 12 months of shared life and having sex without using contraceptive methods), having sexual activity with a spouse during the last three months, no history of chronic diseases (diabetes, hypertension, etc.), infertile women who were infertile for less than 5 years and mental illnesses that affect sexual function, not taking drugs that affect sexual function, lack of allergy to herbs and medicinal plants, and not using tobacco and alcohol. Exclusion criteria were unwillingness to continue the study, not using date palm capsules for three consecutive days, and allergy to date palm capsules during the project.

#### Randomization and blindness

Random allocation method was used as the sampling method. Samples entered the groups alternately. Eligible participants were randomly assigned (1:1) to either the intervention group (A) or the placebo groups (B). We selected the study groups from infertile women referring to the infertility center in Jahrom, who had expressed their consent to participate in the study. Randomization was performed by the Chief Pharmacist. The lead researcher and participants were blinded to the allocation. Participants were not aware of group assignment. The data (Sexual function questionnaires) was collected for two times, before and after the study period.

#### Preparation of date palms and placebo

Date palms were collected from the palm lands of Jahrom, Iran and they were used in the study after collecting them and obtaining the confirmation of experts. Starch powder was applied to prepare the placebo capsules. Date palm and placebo capsules were prepared in similar capsules in terms of consistency, opacity, color, and packaging without any special odor by the Pharmacist. The date palm pollen and placebo capsules were prepared in a pharmacy laboratory of the Faculty of Pharmacy, Jahrom University of Medical Sciences, Jahrom, Iran, by a pharmacist. Each date palm pollen capsule contained 300 mg of date palm pollen and each placebo capsule contained 300 mg of starch. The participants daily consumed one capsule for 30 days. A similar study was used to determine the drug dose and the duration of intervention [21].

#### Intervention

After doing arrangements with the participants, explaining the objectives of the research and signing the consent form, the sexual function questionnaire was handed out to them. Afterwards, the pollen capsules (300 mg daily) and placebo (300 mg daily) were given to the groups to be used daily after breakfast. The participants completed the questionnaire twice, once at the beginning of the study, and once after end of study or placebo with having at least one sexual intercourse.

#### Study questionnaire

The data were collected using the demographic characteristic form and the Female Sexual Function Index Questionnaire and the International Index of Erectile Function.

The International Index of Erectile Function (IIEF) questionnaire was used to assess male sexual function. It consists of 5 areas of erectile function, orgasmic function, sexual desire, intercourse satisfaction and overall satisfaction [22]. In Iran, the sensitivity, specificity and accuracy of this tool has been confirmed with a sensitivity of 8%, specificity of 82%, and positive predictive value of 82% [23].

The Female Sexual Function Index (FSFI) was used to assess female sexual function. It includes 19 items in 6 areas of sexual desire, sexual arousal, lubrication, orgasm, satisfaction and pain. Finally, the total score of sexual function is obtained by summing up of the scores in all areas. It is obtained between 2 and 36, so that a score lower than 26.5 represents sexual dysfunction [24]. It was validated in a research conducted by Mohammadi in Iran (2004). Its total reliability coefficient using the test–retest method was reported to be 75% [25].

#### Statistical analysis

Data were statistically analyzed by SPSS 21 software. Data were analyzed using descriptive statistics (frequency tables, mean and standard deviation) and analytical (independent t-test, chi-square and paired t-test). Significance level was considered 0.05.

### Results

#### Population characteristics

**Fig. 1:**
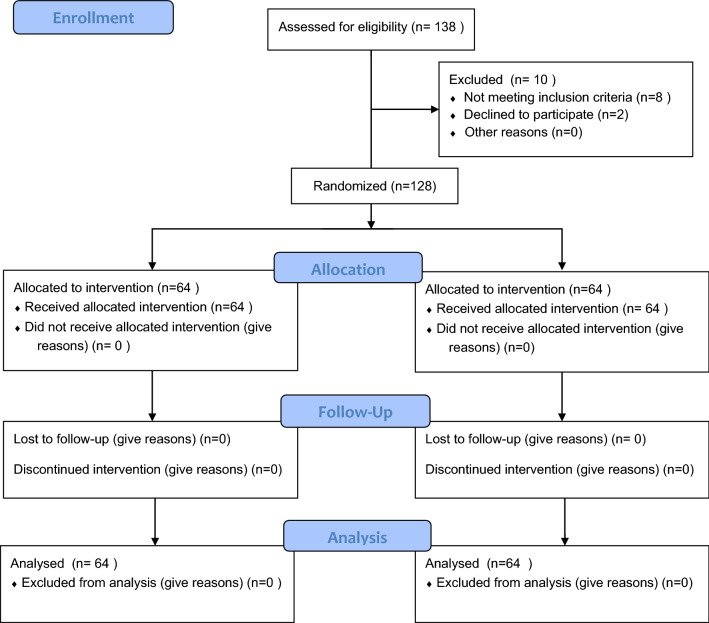
Flow diagram of the study

Among 138 participants examined for eligibility, ten participants withdrew (eight participants did not meet the inclusion criteria and two participants did not complete the questionnaire). A hundred twenty-eight participants met the inclusion criteria that were randomized into intervention (n = 64) or control groups (n = 64). The flow chart of the study enrollment is shown in Fig. 1.

The figure presents the results for the Chi-square test regarding no statistical differences between the two study groups in terms of their employment status, duration of infertility and education level. The mean age of women participating in the control and intervention groups was 29.95 ± 3.88 and 30.01 ± 2.15, respectively (p-value > 0.05) (Table 1).

**Table 1:** Comparison of groups in terms of demographic variables of participants

Variable	Group		p-value
	Intervention group	Control Group	
Woman's age	30.01 ± 2.15	29.95 ± 3.88	0.91
Husband age	34.24 ± 2.20	34.43 ± 4.17	0.75
Employment status			
Housewife	52 (80)	45 (69.2)	0.11
Employed	13 (20)	20 (30.8)	
Educational level			
Primary school	23 (35.4%)	15 (23.1)	0.12
Secondary school	33 (50.8)	33 (50.8)	
Academic	9 (13.8)	17 (26.2)	
Husband job			
Non-employed			0.05
Employed			
Husband educational level			
Primary school	23 (35.4)	18 (27.7)	0.08
Secondary school	34 (52.3)	29 (44.6)	
Academic	8 (12.3)	18 (27.7)	
Duration of infertility			
Tree years	23 (35.4)	28 (43.1)	0.40
Four years	30 (46.2)	30 (46.2)	
Five years	12 (18.5)	7 (10.8)	

P value: Independent t-test, Chi square

The results of the paired t-test for female sexual function indices revealed that the total score of female sexual function in the intervention group increased significantly from 21.06 to 27.31 (P < 0.000). In the intervention group, the mean score of sexual desire increased from 2.92 to 4.75, which was statistically significant (P < 0.0001). Also, other areas of sexual function in women (arousal, orgasm, lubrication, satisfaction) in the intervention group increased significantly compared to women in the control group which was statistically significant (P < 0.0001) (Table 2).

**Table 2:** Comparison of the Sexual function female domains between Control Group and Intervention group (before and after the intervention)

Domains	Intervention group		Control group	p-value**
	Mean ± SD		Mean ± SD	
Libido	Before	2.78 ± 0.74	2.82 ± .95	0.82
	After	4.38 ± 0.71	2.92 ± .79	p < 0.0001
P-value*	p < 0.0001		p = 0.48	
Arousal	Before	2.87 ± 0.37	3.08 ± 1.03	0.12
	After	4.86 ± 0.84	2.86 ± 0.44	p < 0.0001
P-value*	p < 0.0001		p = 0.1	
Orgasm	Before	3.56 ± 1.18	3.90 ± 1.47	0.14
	After	5.15 ± 0.78	3.71 ± 1.16	p < 0.0001
P-value*	p < 0.0001		p = 0.38	
Lubrication	Before	3.47 ± 0.91	3.14 ± 0.98	0.05
	After	4.43 ± 0.79	3.35 ± 1.41	p < 0.0001
P-value*	p < 0.0001		p = 0.29	
Satisfaction	Before	4.49 ± 0.61	4.55 ± 1.35	0.76
	After	5.46 ± 0.67	4.44 ± 0.72	p < 0.0001
P-value*	p < 0.0001		p = 0.55	
Pain	Before	3.86 ± 0.77	3.25 ± .71	0.19
	After	3.00 ± 0.51	3.44 ± 0.74	p < 0.0001
P-value*	p < 0.0001		p = 0.69	
Total score FSFI	Before	21.06 ± 2.58	21.09 ± 6.60	0.97
	After	27.31 ± 2.59	20.74 ± 2.66	p < 0.0001
P-value*	p < 0.0001		p = 0.67	

*P-value: paired t-test

**P-value: Independent t-test

The results of the paired t-test in analyzing erectile function indices of infertile women (men) showed that erectile function increased from 22.32 to 26.98 in the intervention group which was statistically significant. Also, all its sub-areas (orgasm, sexual desire, intercourse satisfaction, overall satisfaction) had a statistically significant increase in the intervention group (Table 3).

**Table 3:** Comparison of men in the two groups in terms of male erectile function index parameters

Domains	Intervention group		Control group	p_value**
	Mean ± SD		Mean ± SD	
Erectile function	Before	22.32 ± 7.39	22.96 ± 4.75	0.55
	After	26.98 ± 3.04	22.43 ± 7.26	p < 0.0001
P-value*	p < 0.0001		0.62	
Orgasmic function	Before	7.50 ± 2.88	7.26 ± 2.19	0.58
	After	9.18 ± 1.40	6.75 ± 2.83	p < 0.0001
P-value*	p < 0.0001		0.16	
Sexual desire	Before	5.84 ± 2.06	5.93 ± 1.36	0.76
	After	8.23 ± 1.55	5.52 ± 1.73	p < 0.0001
P-value*	p < 0.0001		0.14	
Intercourse satisfaction	Before	10.81 ± 3.75	11.81 ± 2.96	0.07
	After	13.13 ± 1.67	11.47 ± 2.32	p < 0.0001
P-value*	p < 0.0001		0.42	
Overall satisfaction	Before	8.69 ± 1.80	8.21 ± 1.32	0.08
	After	9.50 ± 0.86	7.72 ± 1.54	p < 0.0001
P-value*		0.001	0.07	

*P-value: paired t-test

**P-value: Independent t-test

### Discussion

Our results revealed that date palm has a positive impact on sexual function of women and their husbands. The areas of female sexual function (sexual desire, sexual arousal, orgasm, lubrication, satisfaction, and pain) were significantly increased in women treated with date palm compared to women in the control group. This increase was also observed in sexual function parameters of their spouses (erectile function, orgasmic function, sexual desire, intercourse satisfaction and overall satisfaction) compared to the control group. The study conducted by Sadeghi et al. revealed that using date palm in postmenopausal women for 1 month had a positive and significant impact on sexual desire and arousal [21]. Yousefzadeh et al. also reported that using date palm had a positive impact on orgasm, satisfaction and lubrication in women and also reduced pain during intercourse in women [16]. These results were in line with those of our study. Administering date palm to male rats and measuring their sexual behaviors, Abedi showed that sexual behavior parameters (number of ejaculations, number of intercourse) increased compared to the control group [18]. Also, the study conducted by Fallahi et al. in the treatment of male infertility showed that data palm was a very an appropriate supplement for infertility, can reduce free radicals and increase sperm motility [26].

Improvement in male and female sexual function can be due to active ingredients and increased levels of sex hormones following the consumption of date palm [27], since studies indicated that increasing sex hormones is effective in sexual function. They have also referred to role of androgens on female sexual function, especially their sexual desire [28, 29]. Treatment with testosterone and estrogens improves sexual desire, orgasm, sexual arousal, and satisfaction in women with sexual problems [29]. Studies conducted by Shahedaei et al. showed a positive relationship between serum levels of follicle-stimulating hormone and estradiol and areas of sexual desire, sexual arousal, and satisfaction in women [30]. Estradiol also plays a major role in female sexual function, especially in genital tissue survival. Thanks to its vasodilator mechanism, estradiol paves the way for increased vaginal blood flow, leading to blood accumulation in the genitals and vaginal moisture and eventually increased sexual desire [31]. A study conducted by Moshtaghi et al. showed an increase in the concentration of estrogen and progesterone in adult female rats receiving date palm, which could indicate a possible role of date palm in improving sexual function and treating female infertility [32]. It seems that hormones in date palm, such as estradiol, etc., can improve sexual function. Also, the presence of compounds such as alkaloids and flavonoids in dates increases sexual desire and sexual intercourse in males and females by affecting the central nervous system, stimulating dopamine secretion and activating the mesolimbic system and *nucleus accumbens* [27]*.*

### Conclusion

The results of present study revealed that using date palm capsules for 1 month increases the areas of sexual function in males and females infertile without any side effects.

### Limitations

Individual characteristics and mental states, socio-economic issues, family relationships, cultural factors of infertile women can affect sexual function which has not been investigated in this study.

This study was the first to examine the effect of date palm on sexual function of infertile couples. So far no studies has investigated the effect of palm pollen on sexual function in men and women.

Lack of similar human studies in this area made it impossible to compare the results.

It is recommended to conduct studies with longer duration on females and males, especially infertile couples.

Further studies on the use of date palm on sex hormone levels in infertile couples are recommended.

## Data Availability

The datasets generated and/or analyzed during the current research are not publicly available as individual privacy could be compromised but are available from the corresponding author on reasonable request**.**

## Abbreviations

FSFI: Female sexual function index
IIEF: International index of erectile function
